# A 7.4 ps FPGA-Based TDC with a 1024-Unit Measurement Matrix

**DOI:** 10.3390/s17040865

**Published:** 2017-04-14

**Authors:** Min Zhang, Hai Wang, Yan Liu

**Affiliations:** 1 School of Electro-Mechanical Engineering, Xidian University, Xi’an 710071, China; minzhanghk@gmail.com (M.Z.); liuy@xidian.edu.cn (Y.L.); 2 School of Aerospace Science and Technology, Xidian University, Xi’an 710071, China

**Keywords:** time-to-digital converter (TDC), field-programmable gate arrays (FPGA), measurement matrix, delay circuits

## Abstract

In this paper, a high-resolution time-to-digital converter (TDC) based on a field programmable gate array (FPGA) device is proposed and tested. During the implementation, a new architecture of TDC is proposed which consists of a measurement matrix with 1024 units. The utilization of routing resources as the delay elements distinguishes the proposed design from other existing designs, which contributes most to the device insensitivity to variations of temperature and voltage. Experimental results suggest that the measurement resolution is 7.4 ps, and the INL (integral nonlinearity) and DNL (differential nonlinearity) are 11.6 ps and 5.5 ps, which indicates that the proposed TDC offers high performance among the available TDCs. Benefitting from the FPGA platform, the proposed TDC has superiorities in easy implementation, low cost, and short development time.

## 1. Introduction

A time-to-digital converter (TDC) converts time into digital code, and can be regarded as a time sensor. TDC is utilized to measure the time interval or pulse width that corresponds to physical events with high resolution in many applications, such as integrated circuit testing [1,2,3] especially in all-digital phase-locked loop (ADPLL), laser ranging [4], image sensors for fluorescence lifetime imaging (FLIM) [5,6], time-of-flight positron emission tomography (TOF-PET), and high energy physics (HEP) [7,8].

TDC architecture can be implemented in analog or digital approaches. Time stretching and time-to-amplitude conversion methods are two classical analog TDC methods. In the time stretching method, the capacitor is discharged and recharged by two current sources, where the recharging time is *K* times longer than the discharging time. Hence, the time resolution can be improved by the stretching factor [8]. TDC based on a time-to-amplitude conversion method is realized by combining time-to-amplitude conversion and analog-to-digital converters (ADC) [9,10]. Through careful design and layout, analog TDC can obtain good resolution (about 8 ps [10]) at the expense of high power consumption. However, analog TDCs often suffer from large temperature drift and poor stability. Moreover, the area-consuming devices in analog TDC also hinder its implementation in integrated circuits.

With the development of integrated circuit (IC) technologies, recent works have concentrated on all-digital TDCs which employ standard CMOS technology to realize on-chip TDC [11]. Due to their low area consumption and high conversion rate, TDCs based on application-specific integrated circuits (ASIC-based TDC) and field programmable gate arrays (FPGA-based TDC) have been widely used in recent years. Compared with FPGA-based TDC, ASIC-based TDC has the merits of fully customized circuits and precise control of the internal propagation delay [1,2,4,5,6,12,13,14,15,16,17]. The resolution obtained by ASIC-based TDC can be 1–2 ps [1,16]. However, ASIC-based TDCs usually suffer from high development cost and long time-to-market.

Excited by the continued development in semiconductor technology, FPGA devices have become very popular for rapid system prototyping, logic emulation, and reconfigurable computing because of their much lower manufacturing cost and shorter development time. In recent years, several high-performance TDCs based on FPGAs have been reported [18,19,20,21,22,23,24,25,26]. Most of the FPGA-based TDCs use vernier delay lines (VDLs) or tapped delay lines (TDLs) based on carry logics [18,23,24,25,26], digital delay elements [19,21], buffers, or other integrated delay elements [20]. However, the internal propagation delay of FPGA-based TDC cannot be fully customized, which significantly limits the time resolution. The best time resolution of previous FPGA-based TDCs is about 10 ps [20,22,24,25].

Most FPGA-based and ASIC-based TDCs use delay lines built by special delay elements. However, realizing a high-linearity delay line in FPGA devices is a great challenge, due to the innate delay differences. Another challenging problem for both ASIC-based and FPGA-based TDCs is that they are also sensitive to voltage and temperature variations, which decreases the measurement stability.

In this paper, a novel architecture for FPGA-based TDC with a 1024-unit measurement matrix and parallel delay elements is proposed and tested. The most important advantage of the proposed TDC relies on the routing resources used to build the delay elements that are different from the existing designs and proves less sensitive to variations of temperature and voltage. In the FPGA device, routing resources are mainly copper wires with nanometer manufacturing process, which is insensitive to the drift of operating voltage and temperature. Moreover, the proposed method provides a new application for counter method, which has been used only in “coarse” measurement in recent years. Experimental results indicate the high performance of the TDC.

This paper is organized as follows. In Section 2, the principle of the proposed TDC is described. Section 3 introduces the architecture and implementation of the TDC. The evaluation system and the experimental results are presented in Section 4. Finally, Section 5 summarizes and concludes the paper.

## 2. Principle of the Proposed TDC

### 2.1. Counter Method

**Figure 1:**
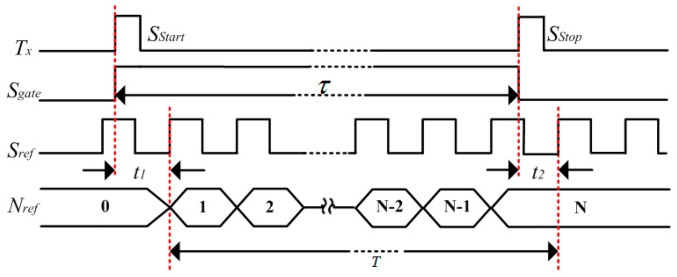
Timing diagram of the counter method.

Counter method is one of the most classical digital TDC methods, and counts the time interval with the cycle time of a reference clock. The timing diagram of a counter method is shown in Figure 1. In the figure, the time interval is represented by $T_{x}$, and the gate signal ($S_{\mathrm{gate}}$) is generated according to $T_{x}$. The rising edge of $S_{\mathrm{gate}}$ enables the counter, while the falling edge disables the counter. $S_{\mathrm{ref}}$ is the reference clock, with a frequency of $f_{\mathrm{ref}}$. The measured time interval can be calculated by Equation (1).

(1) $$T_{x}\;=\;\frac{N_{\mathrm{ref}}}{f_{\mathrm{ref}}}$$

(2) $$\Delta T_{x}\;=\;T\;–\;\tau \;=\;t_{2}\;–\;t_{1}$$

The measurement error ($\Delta T_{x}$) of the counter method can be calculated from Equation (2), which is within the range of $\left(–\frac{1}{f_{\mathrm{ref}}},\;\frac{1}{f_{\mathrm{ref}}}\right)$. The time resolution of the counter method is limited to several nanoseconds, hindered by the frequency of the reference clock. The counter method is usually used to be the “coarse” time digitizing method in recent works [13,24,25], and is employed to enlarge the measurement range.

### 2.2. Counter Method Based on Delay Line 

**Figure 2:**
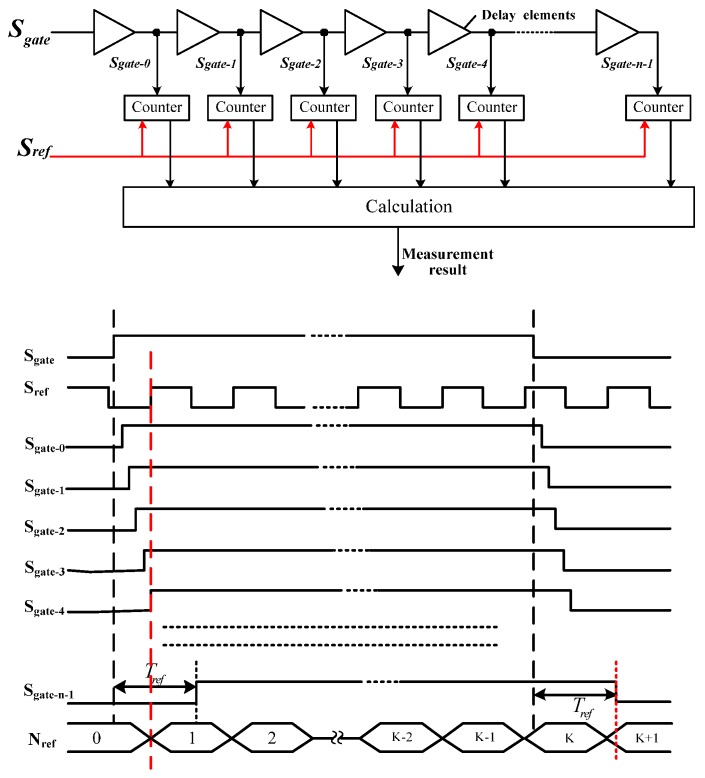
Structure and timing diagram of improved counter method based on delay line.

To improve the time resolution, a modified counter method based on delay line is proposed. The measured time interval signal is delayed through a delay line, and gate signals with different delays are generated. The structure and timing diagram of the modified method is shown in Figure 2. The original gate signal and its delayed versions form a group of uniformly delayed gate signals. Each signal in the group is counted by the same reference clock. Then, the measurement result and measurement error can be calculated from Equations (3) and (4), where $N_{\mathrm{ref}.i}$ represents the output of the counters:

(3) $$T_{x}\;=\;\frac{\frac{1}{n}\sum_{i=0}^{n–1}N_{\mathrm{ref}.i}}{f_{\mathrm{ref}}}$$

(4) $$\Delta T_{x}=\frac{1}{n}\sum_{i=0}^{n-1}\Delta T_{x.i}=\frac{1}{n}\sum_{i=0}^{n-1}(t_{2.i}-t_{1.i})$$

The counter method based on a delay line requires that the delay of delay elements in the line satisfy Equation (5), where $D_{i}$ represents the time delay of delay elements and *D* is a constant. Then, $\Delta T_{x.i}$ and $\overset{n–1}{\underset{i=0}{\sum }}\Delta T_{x.i}$ can be expressed as the equations below, where the function fmod(A,B) returns the remainder after division of *A* by *B.*

(5) $$D_{i}\;=\;\frac{1}{{n\;\times \;f}_{\mathrm{ref}}}\;=\;D,\;i\;=\;1,\ldots ,n\;–\;1$$

(6) $$\{\begin{array}{c} t_{1.i}\;=\;\mathrm{fmod}(\left(t_{1.0}\;+\;i\;\times \;D\right),\;\frac{1}{f_{\mathrm{ref}}})\\ t_{2.i}\;=\;\mathrm{fmod}(\left(t_{2.0}\;+\;i\;\times \;D\right),\;\frac{1}{f_{\mathrm{ref}}}) \end{array}$$

(7) $$\Delta T_{x.i}{\;=\;t}_{2.i}\;–\;t_{1.i}\;=\;\mathrm{fmod}\left(\left(t_{2.0}+\;i\;\times \;D\right),\;\frac{1}{f_{\mathrm{ref}}}\right)\;–\;\mathrm{fmod}(\left(t_{1.0\;}+\;i\;\times \;D\right),\;\frac{1}{f_{\mathrm{ref}}})$$

(8) $$\sum_{i=0}^{n\;–\;1}\Delta T_{x.i}=\sum_{i=0}^{n–1}t_{2.i}-\sum_{i=0}^{n–1}t_{1.i}$$

$\left\{t_{1.i},\;i\;=\;0,\;1,\;2,\;3,\ldots n\;–\;1\right\}$ and $\left\{t_{2.i},\;i\;=\;0,\;1,\;2,\;3,\ldots n\;–\;1\right\}$are both arithmetic sequences with a common difference of D, which are obtained by realigning $\left\{t_{j.i},\;j\;=\;1,\;2;\;\right\}$ according to the values. According to the summation formula of arithmetic sequence, $\overset{n–1}{\underset{i=0}{\sum }}t_{j.i}$ can be estimated by Equation (9). The measurement error can be estimated by Equation (10), which is reduced to 1/*n* of the original value. Therefore, the improved counter method reduces the measurement error effectively, attributed to the time delay of delay elements instead of the cycle time of a reference clock. Additionally, this method delayed the gate signal instead of the reference clock [13,24], which is much easier to implement.

(9) $$\frac{n\;-\;1}{2}\;\times \;\frac{1}{f_{\mathrm{ref}}}\leq \overset{n\;–\;1}{\underset{i=0}{\sum }}t_{j.i}\;<\frac{\;n\;+\;1}{2}\;\times \;\frac{1}{f_{\mathrm{ref}}},\;\left(j=1,2;\;i=0,1,2,3,\ldots n-1\right)\;$$

(10) $$–\frac{1}{{n\;\times \;f}_{\mathrm{ref}}}\;<\Delta T_{x}\;=\;\frac{1}{n}\sum_{i=0}^{n–1}\Delta T_{x.i}\;<\frac{1}{{n\;\times \;f}_{\mathrm{ref}}}$$

### 2.3. Counter Method Based on Parallel Delay Elements

For the improved counter method, the difficulty is that the delay linearity of the delay line must satisfy Equation (5), which contains two constraints. One is that the delay of the line should equal to the cycle time of reference clock ($\frac{1}{f_{\mathrm{ref}}}$). The other is that the delay line must have a good delay linearity, which means the delay of each delay element should be equal to $\frac{1}{{n\;\times \;f}_{\mathrm{ref}}}$.

Inside an FPGA chip, logic gates, buffers, and carry logics can all be employed to construct the delay line shown in Figure 2. Most of them cannot provide good delay linearity due to the innate delay differences, which is mainly caused by the unpredictable P&R (Place and Route) delay in the process of implementation. However, carry logic is an exception. In order to perform fast arithmetic addition and subtraction operations in an FPGA device, carry logics are equipped with independent dedicated logic elements and routing paths, the delay of which increase linearly with the number of bits in the operand. Delay lines built by carry logics can provide good delay linearity [18,22,23,24]. Therefore, FPGA-based TDCs utilizing carry logics can achieve a good time resolution of about 10 ps.

A novel implementation of delay line based on an FPGA chip is proposed in [20], which uses routing paths as the delay elements and achieves good delay linearity. A practical disadvantage of the method is that much time and manpower are consumed in adjusting the routing of propagation paths. However, the FPGA device is abundant in routing resources, and using routing paths as the delay element is still interesting and promising.

**Figure 3:**
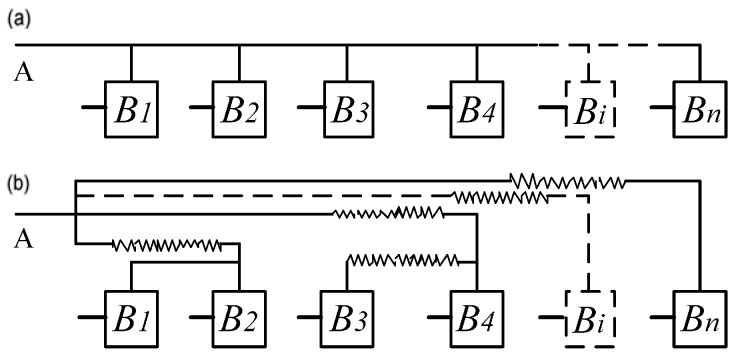
Architecture of propagation path: (**a**) architecture of propagation path needed in ideal conditions; (**b**) architecture obtained in default routing mode.

**Figure 4:**
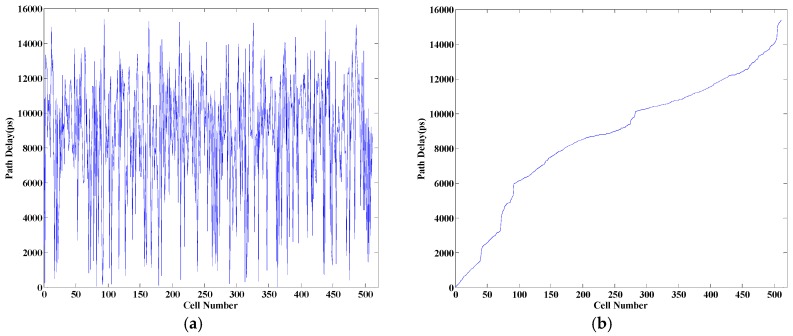
Figure of delay characteristic of the propagation paths: (**a**) delay characteristic of the obtained propagation paths; (**b**) delay characteristic of the 512 paths according to the delay values.

The FPGA placer and router not only bring unpredictable delay to the circuit, but also always implement the circuit shown in Figure 3a into the circuit shown in Figure 3b, where B_1_~B*n* are logic cells. In Figure 3a, the ideal propagation path is designed according to Equation (11). However, the obtained delay characteristic is shown in Figure 4a, which is obtained when *n* is 512. In Figure 4a, the delay characteristic of the 512 propagation paths is messy without regular rules. However, Equation (11) requires that the delay values of the 512 propagation paths have good delay linearity, and the structure or the sequence of the paths is unconstrained. Figure 4b can be obtained by rearranging the path delays shown in Figure 4a according to the delay values, which denotes a characteristic similar to the linear increase.

(11) $$P_{{A–B}_{2}}\;–\;P_{{A–B}_{1}}{=\;P}_{{A–B}_{3}}\;–\;P_{{A–B}_{2}}{\;=\;P}_{{A–B}_{\left(i+1\right)}}\;–\;P_{{A–B}_{i}}\;,\;(i\;=\;1,\;2,\;3,\;\ldots \;n\;–\;1)$$

**Figure 5:**
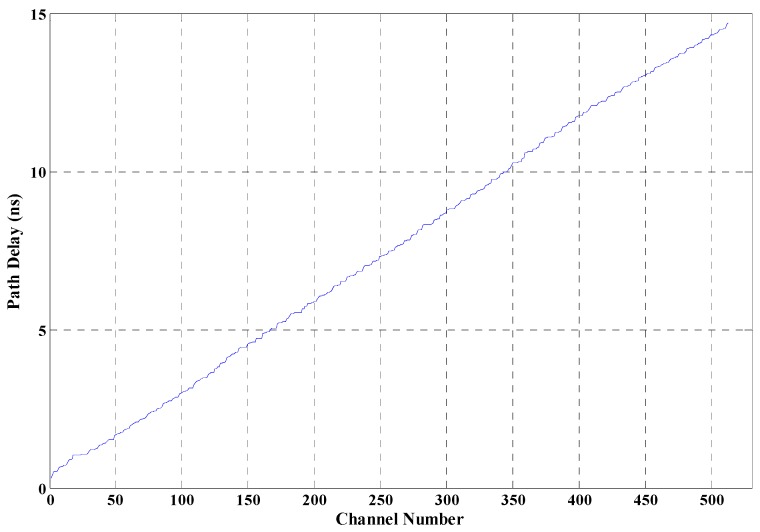
Delay characteristic of the 512 routing paths after conducting adjustments.

The linearity shown in Figure 4b is not good, and experimental results suggest that a random error in the delay linearity less than 10 percent would lead to about 50 percent decrease in time resolution. Further research denoted that the path delays shown in Figure 4b could be improved to what is shown in Figure 5 after performing adjustments by defining the location and structure of propagation paths, which is described in detail in Section 3. Hence, this paper considers utilizing the routing paths to be the delay elements and constructs a time-to-digital converter system. For the delay elements that are parallel, the method is named as “measurement matrix based on parallel delay elements”.

## 3. Implementation of the Proposed TDC

In Figure 5, 512 routing paths are built, and the time delay of the 512 paths are uniformly distributed in the range of 313 ps to 14,683 ps. Hence, the theoretical time resolution is (14,683 − 313)/512 ≈ 28.07 ps. In order to obtain better resolution, a TDC based on a measurement matrix with 1024 parallel delay elements and implemented in the Xilinx FPGA chip is researched.

**Figure 6:**
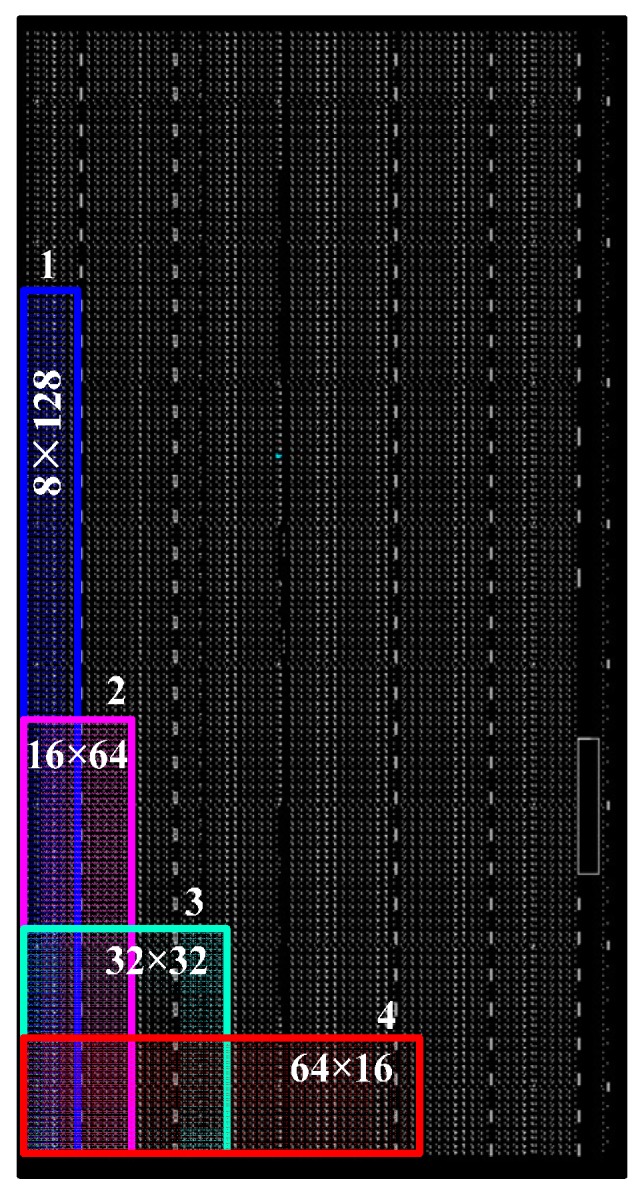
Structures of the constraint area.

**Figure 7:**
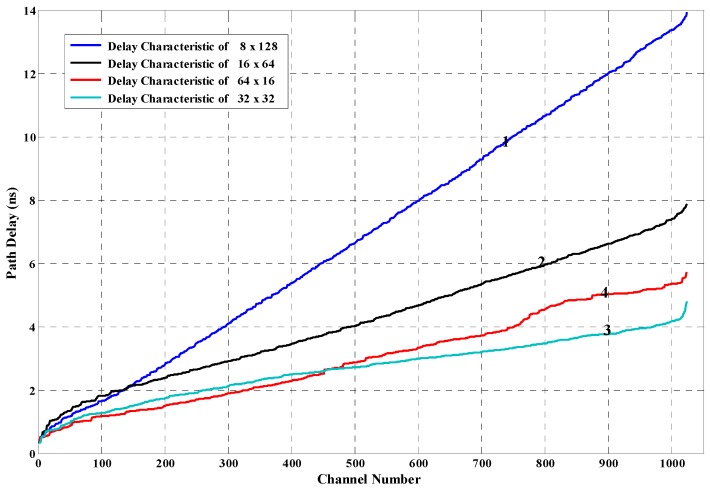
Delay characteristic of the 1024 routing paths under different structures of the constraint area.

The path delay shown in Figure 4a cannot be used to implement the TDC system, because the default placing and routing conducted by the FPGA placer and router cannot provide good delay linearity. Adjustments are performed in two aspects to improve the delay linearity. First, constraints are performed to restrain the location and the shape of the measurement matrix. The counter of a measurement unit is restrained into a slice, which is a basic logic cell of the Xilinx FPGA device. Inside the FPGA, logic cells are identified with plane coordinates. This paper restrained the shape of the 1024 measurement matrix, which can be restrained in structures of eight logic cells in width (Slice X0Y0~X7Y0) and 128 logic cells in length (Slice X0Y0~X0Y127), represented by 8 × 128. Figure 6 shows the distribution of several structures, 8 × 128, 64 × 16, 32 × 32, and 64 × 16. The delay characteristic of the 1024 units under different structures is shown in Figure 7, which illustrates the influences of different shapes of the measurement matrix to the delay characteristic. In Figure 7, line 1—corresponding with the shape 8 × 128—has the best delay linearity. Second, the routing order of signals is also changed manually. The network of gate signal is routed first, and then we route the network of reference clock. After that, we route the rest of the signals. Compared with Figure 4, Figure 5 is obtained by conducting the adjustments above and restraining the shape of the measurement matrix as 8 × 64, which suggests that the adjustments are highly effective. Compared with the manual adjustments performed in [20], which need to adjust each routing path manually and are very time-consuming, the adjustment in this paper is much simpler and easier.

**Figure 8:**
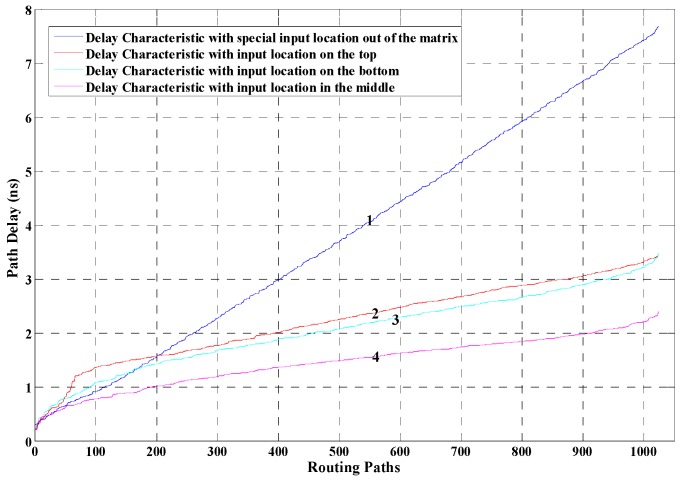
Delay characteristic of the 1024 routing paths under different input locations of the measured signal.

Moreover, during the implementation, we find that the delay characteristic of the 1024 paths is also influenced by the input location of the to-be-measured time interval signal. The input location can choose from all the unused area of the chip, which provides us with great implementation flexibilities. In this paper, different input locations of time interval signal are evaluated. Figure 8 is obtained under the condition that the shape of the constraint area is 8 × 128. In the figure, line 1 denotes the delay characteristic with special input location out of the measurement matrix, while line 2, line 3, and line 4 denote the delay characteristic with the input location on the top, at the bottom, and in the middle of the measurement matrix.

In order to choose the structure that provides the best resolution and linearity, the delay characteristic of 1024 routing paths under different circumstances have been carefully analyzed and compared. For a 1024-unit measurement matrix, range of the time delay of the 1024 paths determines the time resolution, while the delay linearity of the 1024 paths decides the linearity of the TDC. The linearity of the lines shown in Figure 7 and Figure 8 are calculated and compared. Calculation results of linearity prove that the 8 × 128 structure with special location constraints for the input time interval signal (line 1 in Figure 8) achieves the best delay linearity. Although some of the lines shown in the figures can provide higher theoretical time resolution (e.g., line 4 in Figure 7 and Figure 8), their delay linearities are not good and the measurement errors are distributed unevenly. On the other hand, according to Figure 2, the cycle time of the reference clock should be equal to the delay difference between the maximum delay and the minimum delay of the 1024 paths. Thus, the smaller the delay difference is, the higher the frequency of the reference signal should be. For example, for line 4 in Figure 8, the largest delay difference of the paths is less than 2.5 ns, and the frequency of the reference signal should be higher than 400 MHz. However, high frequency with a large number of fan-outs will bring unpredictable skews and jitter into the circuit, which largely decreases the measurement stability.

After analysis and experiments, this paper implements the 1024-unit measurement matrix based on line 1 in Figure 8. The maximum path delay is 7678 ps, and the minimum path delay is 302 ps. Therefore, the theoretical time resolution is (7678 − 302)/1024 ≈ 7.2 ps. The frequency of the reference signal should be 135.5 MHz.

**Figure 9:**
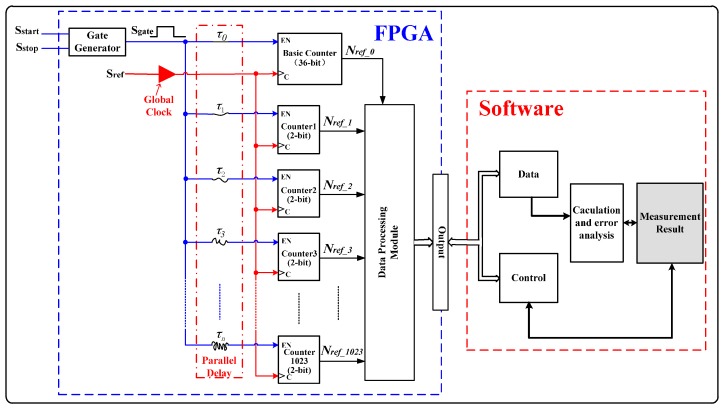
Diagram of a time-to-digital converter (TDC) measurement matrix with 1024 units. FPGA: field programmable gate array.

**Figure 10:**
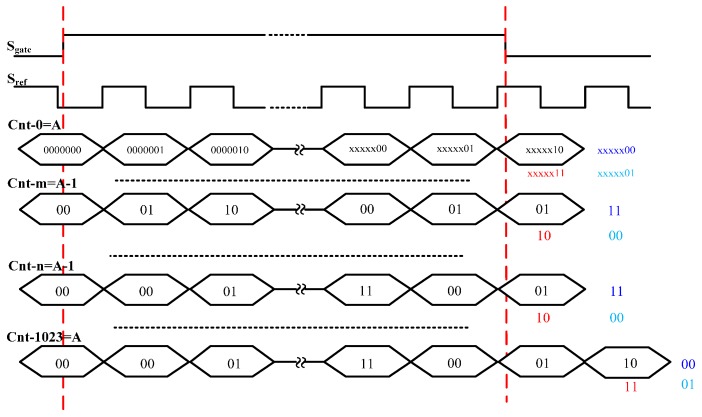
Calculation principle of the 2-bit counters.

The diagram of 1024-unit TDC measurement matrix is shown in Figure 9. $\tau_{0}\sim \tau_{1023}\;(\tau_{0}<\tau_{1023})$ are the delay of the 1024 paths. Unit-0 uses a 36-bit counter, while other units only use 2-bit counters. The 36-bit gray-code counter is used to entirely count the rising edges of the reference clock within the measured time interval, which decides the measurement range of the TDC. According to the principle of the proposed method shown in Figure 2, the difference in counting result between unit-1 to unit-1023 and unit-0 are either 0 or ±1. Therefore, only a 2-bit counter is needed for unit-1 to unit-1023, which greatly decreases the utilization of logic resources. Figure 10 shows one situation of the calculating principle of the counters, where the difference in counting result between unit-1 to unit-1023 and unit-0 is 0 and −1. The output of all the counters is calculated by the data processing module, which is utilized to calculate the measurement result according to Equation (3). The measurement result can be obtained at the output of the data processing module. In this design, large measurement range and high resolution can be obtained by single measurement, while some TDC methods used the two-stage (“coarse and fine”) architecture to enlarge the measurement range [12,17], which directly decreases the measurement accuracy.

**Figure 11:**
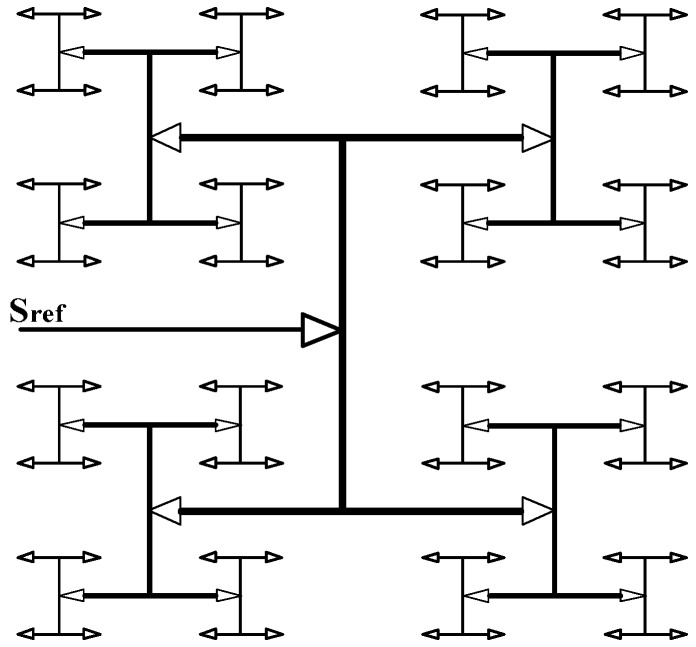
Structure of global clock tree and net.

In Figure 9, the reference clock (135.5 MHz) is fed into the specific input pin of the global clock in an FPGA chip, which drives a global clock buffer. The structure-special and fully-buffered global clock distribution network shown in Figure 11 is used to decrease the clock skew caused by the differences of path and loading. Global clocks (GCLK) are a dedicated network of interconnection that is specifically designed to reach all clock inputs to the various resources in an FPGA [27]. In this system, the reference clock is input into all the measurement units. If the propagation path of the reference clock has large skew or the arriving time of the rising edge of the reference clock is unsynchronized, the time resolution and measurement error will be affected. The largest time difference in the rising edge of the reference clock using GCLK is 107 ps, while that using regional clocking resources is 410 ps. By feeding the reference clock into GCLK, the rising edge of the reference clock to each measurement unit is almost synchronized, and the skew and jitter of the reference clock is largely decreased.

## 4. Experiments and Results

**Figure 12:**
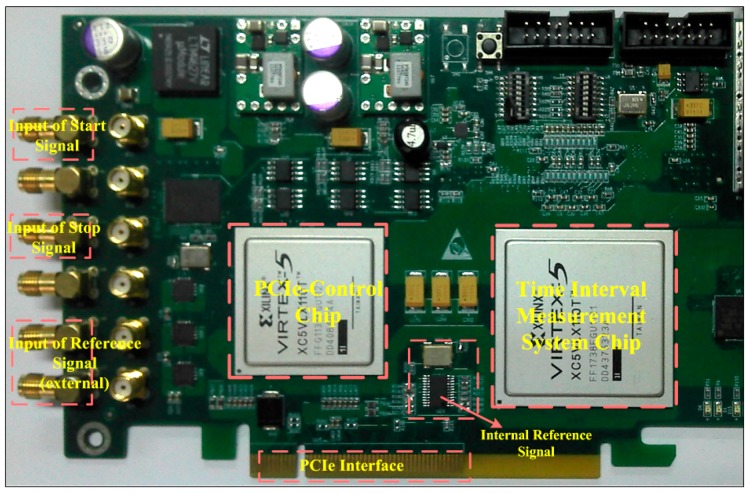
Evaluation board of the proposed FPGA-Based TDC.

**Figure 13:**
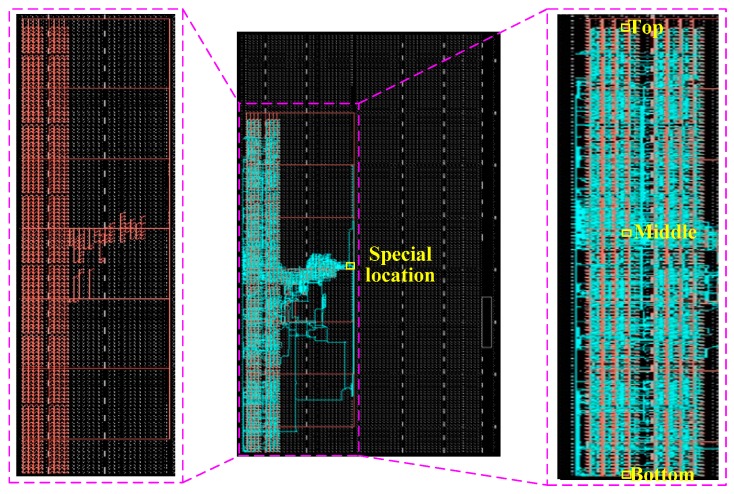
Place and route of the TDC inside the FPGA chip.

To evaluate the performance of the proposed system, the design was implemented on an evaluation board equipped with a Xilinx Virtex-5 XC5VLX110T FPGA (Xilinx Inc., San Jose, CA, USA), shown in Figure 12. The architecture and the circuit of the TDC inside the chip are shown in Figure 13, which was obtained from the Xilinx FPGA Editor (a design tool of ISE design suite). In the figure, the red line (on the left side) is the net of the reference clock, the cyan line (on the right side) is the net of the gate signal, and the input locations of the time interval signal are marked yellow in the figure. It can be seen that the nets of the reference signal and gate signal have good uniformity within the constraint area. The utilization summary and power analysis of the proposed TDC is summarized in Table 1, which indicates that the utilization of logics and resources is low. The utilization summary of the 512-unit TDC is also provided in the table, which is about half of the utilization of the 1024-unit TDC. The dynamic power consumption of the 1024-unit TDC was only 23 mW (obtained through the Xilinx XPower Analyzer, a design tool of ISE design suite), which was obtained by eliminating the quiescent power consumption of the chip.

**Figure 14:**
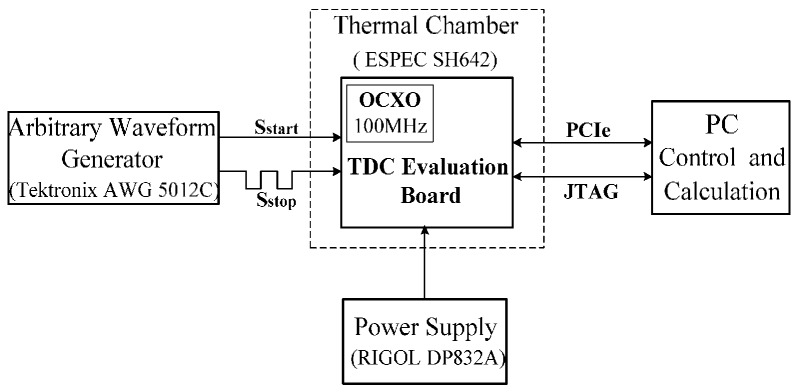
Test system of the TDC. OCXO: oven-controlled crystal oscillator.

The test bench of the TDC is shown in Figure 14. An arbitrary waveform generator (Tektronix AWG 5012C, Beaverton, OR, USA) was employed to generate a time signal. A time interval was created by passing the generated time signal through a resistive power splitter and coaxial cables with different lengths. For example, a constant time interval of 0.5 ns equals approximately 10 cm cable length difference. During the experiments, cable length differences from 10 cm to 3.5 m were used. An OCXO (oven-controlled crystal oscillator) with a frequency of 100 MHz was used for the input clock of the FPGA, and the reference clock was generated by the PLL (phase-locked loop) module in the FPGA. The power of the board was supplied by a RIGOL DP832A (RIGOL Technologies Inc., Beijing, China). Temperature test was performed by using a thermal chamber from ESPEC Corp (ESPEC Corp., Osaka, Japan). The TDC evaluation board communicates with the PC via PCI express.

### 4.1. Time Resolution of the TDC

Experiments were carried out with a nominal supply voltage and at an ambient temperature of about 25 °C. The number of active measurement units ranged from 0 to 1023. When the input time interval was 3000 ps (about 60 cm cable length difference, marked as T_1_), the summation of all of the 1024 counters was 57 (Cnt_1_). When the input time interval was 17,000 ps (about 3.4 m cable length difference, marked as T_2_), the summation of all the 1024 counters was 1948 (Cnt_2_). Therefore, the time resolution was 7.4 ps, which is calculated by Equation (12):

(12) $$R_{\mathrm{LSB}}{\;=\;(T}_{2}\;–\;T_{1}{)/(\mathrm{Cnt}}_{2}\;–\;{\mathrm{Cnt}}_{1})$$

Moreover, the conversion rate of the proposed TDC was also measured. Experimental results indicate that the sample and conversion time of the TDC was 8–11 periods (about 80 ns) of the reference clock, which means the conversion rate of the TDC was about 12.5 Msps.

### 4.2. Nonlinearity of the TDC

**Figure 15:**
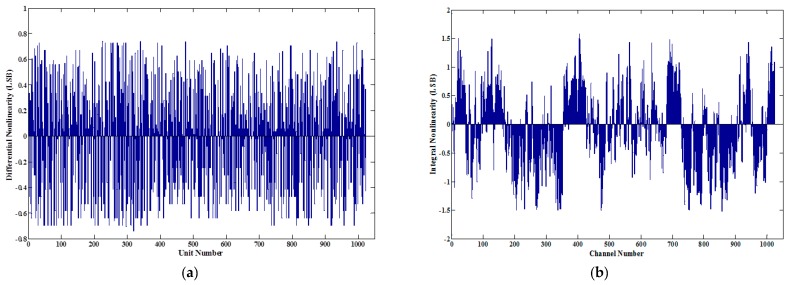
Nonlinearity of the TDC: (**a**) differential nonlinearities (DNL) and (**b**) integral nonlinearities (INL) of the system obtained from a series of 200,000 measurements of random pulses.

In order to test the nonlinearity of the proposed TDC, the well-known statistical code density test method was used. For the code density test, time intervals with random widths were input to the TDC to be tested, and a large number of measurements were performed. A histogram of the output codes can be obtained by the measurements, which presents the number of times that registers in each output code. Dividing the number of the corresponding codes registered by the total number of samples, the bin width of each code can be acquired. Then, the nonlinearity was obtained by calculating the variations of the bin widths [8,28]. In the test, 200,000 samples are acquired. The measurement results were sorted into 1024 bins, and the differential nonlinearities (DNL) and integral nonlinearities (INL) were obtained by calculating the number of counts in each bin. The obtained DNL and INL are shown in Figure 15. The DNL was between −0.74 to +0.74 LSB (5.5 ps), and the INL was within the range of –1.52 to 1.57 LSB (11.6 ps).

The nonlinearity of the TDC was mainly caused by the time delay of the 1024 paths, which can be denoted by the nonlinearity within the range of 0–7.58 ns. When the time interval was larger, the counting result of unit-0 increased with the increasing of the time interval. The results of counters in unit-1 to unit-1023 were calculated according to their counting results and the counting result of unit-0. Therefore, the linearity within the range of 0–7.58 ns can represent the linearity of the proposed TDC within the whole measurement range.

### 4.3. Jitter of the TDC

#### 4.3.1. Standard Deviations of the TDC 

**Figure 16:**
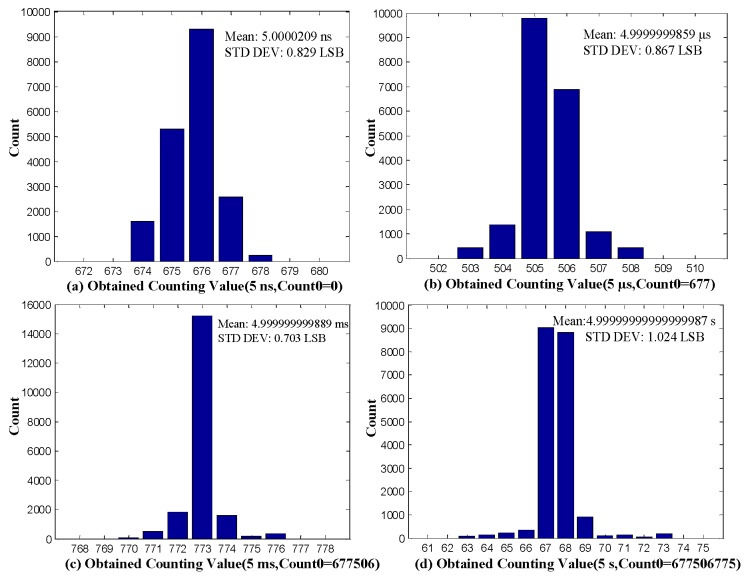
Distributions of time interval measurements obtained for four constant time intervals.

To evaluate the jitter of the proposed TDC, constant time intervals of 5 ns, 5 µs, 5 ms, and 5 s were measured. The 5 ns time interval was obtained by passing a signal from the waveform generator through a resistive power splitter and cables with different lengths. The longer time intervals were generated by the waveform generator, which can generate time intervals within the range of 1 µs–10 s. Each time interval was measured 20,000 times, and the distributions of measurement results are shown in Figure 16. The achieved standard deviations (STD DEV) are, respectively, 0.829 LSB (6.1 ps), 0.867 LSB (6.4 ps), 0.703 LSB (5.2 ps), and 1.024 LSB (7.6 ps). Standard deviation is calculated from Equation (13), where σ is the standard deviation, $X_{i}$ is the measurement result of the *i*th time, and N is the measurement times. During the STD DEV measurement, the input to the TDC is kept fixed. Hence, the STD DEV presents the single-shot precision of the proposed TDC [8].

(13) $$\sigma =\frac{1}{\sqrt{N-1}}\sqrt{\sum_{i=1}^{N}{\left(X_{i}-\frac{\sum_{i=1}^{N}X_{i}}{N}\right)}^{2}}$$

**Figure 17:**
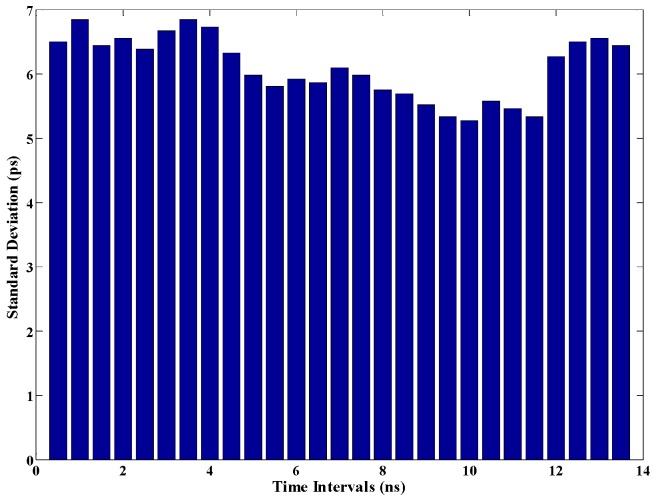
Standard deviation under different time intervals.

The realized TDC was also evaluated for time intervals with the range of 0.5–13.5 ns, and each time interval was measured 1000 times. The standard deviation for each time interval is shown in Figure 17. It can be seen from the figure that the performance is stable with the range of 0.5–13.5 ns. The measurement range was decided by the bit width of the counter in unit-0. The measurement uncertainty shown in Figure 17 covers a cycle time of the reference clock, and can indicate the stability of the system.

#### 4.3.2. Phase Noise of the Reference Clock

The phase noise of the reference clock was measured through Keysight X-Series Signal Analyzer (Keysight PXA N9030A, Santa Rosa, CA, USA). The period jitter of the reference clock can be calculated from Equation (14), where *L*(*f*) is the phase noise. The results of phase noise are shown in Table 2. Moreover, Table 2 also compares phase noise of the reference clock generated by PLL and digital clock manager (DCM). The DCM contains a delay-locked loop (DLL) to provide clock management features. The results show that the reference clock generated by PLL has better performance than that generated by the DCM.

(14) $$\begin{array}{c} RMS\;J_{Period}=\frac{1}{2\pi f_{c}}\sqrt{2\overset{N-1}{\underset{i=1}{\sum }}10^{\frac{b_{i}}{10}}f_{i}^{\frac{-a_{i}}{10}}{\left(\frac{a_{i}}{10}+1\right)}^{-1}\left[\left(f_{i+1}^{\frac{a_{i}}{10}+1}\right)-\left(f_{i}^{\frac{a_{i}}{10}+1}\right)\right]}\\ a_{i}=\frac{\left[L\left(f_{i+1}\right)-L\left(f_{i}\right)\right]}{\left[log\left(f_{i+1}\right)-log\left(f_{i}\right)\right]},\;b_{i}=L\left(f_{i}\right) \end{array}$$

### 4.4. Temperature and Voltage Sensitivity

**Figure 18:**
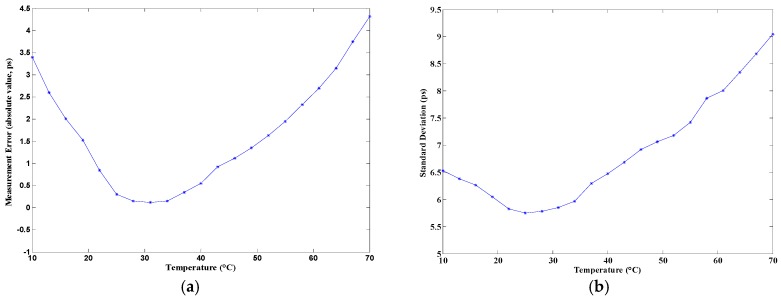
Temperature sensitivity: (**a**) Measurement error and (**b**) Standard deviation of the proposed TDC.

The temperature sensitivity of the proposed TDC was tested on the evaluation board. The temperature tests were performed with the use of a thermal chamber. A constant time interval (5 ns) was measured under different temperatures, with a range from 10 °C to 70 °C. The measurement error under different temperatures—which was obtained by taking the average of the 50 measurement results—is shown in Figure 18a. In the figure, the largest variation in measurement error was 4.3 ps. The standard deviation under different temperatures is shown in Figure 18b, where the largest standard deviation was about 9 ps. The temperature curves of measurement error and standard deviation indicate that the standard deviation increases with the increase of temperature in the range of 25 °C to 70 °C, and the TDC has the best performance around normal temperature (25 °C to 35 °C).

**Figure 19:**
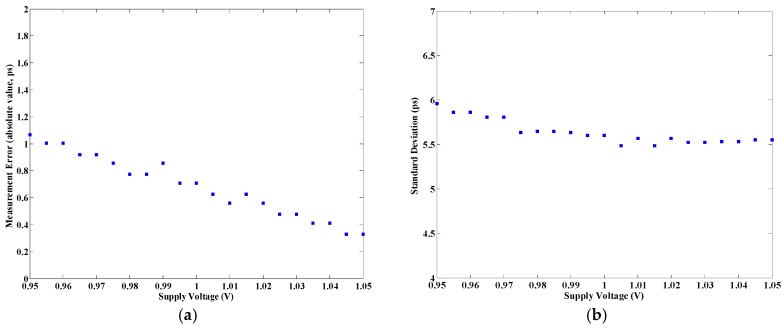
Voltage sensitivity: (**a**) Measurement error and (**b**) Standard deviation of the proposed TDC.

The voltage tests were performed using the RIGOL DP832A DC power supply. The nominal operating voltage of the TDC board is 1 V (internal supply voltage of FPGA). In the tests, the supply voltage of the FPGA was changed within the range of 0.95 V to 1.05 V, differed by 5 mV. A constant time interval (5 ns) was measured under different supply voltages at an ambient temperature of about 25 °C. Measurement under a voltage was repeated 50 times. The measurement error and standard deviation of the measurement results are shown in Figure 19. In the figure, with the change of supply voltage, the largest variation in measurement error and standard deviation were both less than 1 ps, which indicates that the proposed TDC was insensitive to variations of supply voltage.

Compared with other TDCs, the proposed TDC was less sensitive to temperature [24,25] and voltage variations. The temperature stability of the proposed TDC benefitted from the utilization of routing paths. The routing resources inside the FPGA are mainly realized by metal lines (copper), which are not sensitive to normal temperature and voltage variations. However, semiconductor devices such as transistors and diodes inside the FPGA are more sensitive to changes of temperature. In this paper, the temperature drift was mainly caused by the counters. As for the voltage sensitivity, the threshold voltage of the transistors in Xilinx FPGA is about 100 mV [29], and the supply voltage was much larger than the threshold voltage. When the supply voltage is about 1 V, the influence of variations of voltage on the circuit is very small.

### 4.5. Comparisons with Other TDCs

Table 3 compares the performance of the proposed TDC with those of other FPGA-based TDCs presented in the literature. The table indicates that the proposed TDC shows a DNL of 5.5 ps and a time resolution of 7.4 ps. Additionally, this work proposes a novel measurement architecture and implementation method, while other works in the tables are all based on delay lines. Compared with the TDCs in the table, the proposed TDC provides the best resolution, which is similar performance with ASIC-based TDCs.

## 5. Conclusions

An FPGA-based TDC is proposed and tested in this paper. The obtained resolution was 7.4 ps, and the DNL and INL were 5.5 ps and 11.6 ps, respectively. Experimental results prove that the proposed TDC provides higher performance in temperature and voltage sensitivity.

This paper realized a 1024-unit TDC measurement matrix, which introduced a new architecture for TDC implementation. The proposed method may usher in a new application for the counter method, which in recent years has mostly been used only in “coarse” measurement. Taking advantage of the FPGA devices, the proposed TDC features ease of implementation, low cost, and short development time. Moreover, the proposed TDC is portable and can be easily implemented on most general-purpose FPGA platforms. A TDC measurement matrix implemented on the FPGA platforms with better process (28 nm, 20 nm, and 16 nm) is very promising to realize sub-picosecond-resolution FPGA-based TDCs.

## Figures and Tables

**Table 1:** Device utilization summary and power consumption.

		1024-Unit TDC		512-Unit TDC	
	Available	Used	Utilization	Used	Utilization
Slice Registers	69,120	1410	2%	602	0.9%
Slice LUTs	69,120	666	1%	327	0.5%
Occupied Slices	17,280	1265	7%	652	3.7%
Bonded IOBs	640	25	3%	25	3%
Block RAM/FIFO	148	2	1%	2	1%
Clock Resources	32	4	12%	2	6%
Number of routed lines		13,127		4937	
Dynamic Power Consumption		23 mW		9 mW	
Total Power Consumption		1.113 W		1.087 W	

**Table 2:** Phase noise of the reference clock generated by phase-locked loop (PLL). DCM: digital clock manager.

Frequency (Hz)	Phase Noise (dBc/Hz, PLL)	Phase Noise (dBc/Hz, DCM)
100	−62.66	−56.83
1000	−107.48	−85.06
10,000	−115.43	−93.08
100,000	−119.56	−92.03
1000,000	−120.05	−103.25

**Table 3:** Performance summary and comparison to previous works.

Work	This Work	[19] (2009)	[20] (2009)	[21] (2011)	[22] (2013)	[23] (2014)	[24] (2016)	[25] (2015)
Principle	Matrix of counters	Vernier delay cells	Vernier delay line	Multiple delay line	Tapped delay line	CARRY delay line	Tapped delay line	Tapped delay line
Processing Technology	65 nm	90 nm	65 nm	0.15 µm	65 nm	90 nm	40 nm	28 nm
Number of Bins	1024	200	120	220	256	110	128	80
Resolution	7.4 ps	75 ps	11 ps	50 ps	15 ps (RMS)	20 ps	10 ps	15 ps (RMS)
DNL	0.74 LSB	2.5 LSB	0.33 LSB	1.9 LSB	3 LSB	-	1.91 LSB	1 LSB
INL	1.57 LSB	2.8 LSB	0.8 LSB	2.2 LSB	4 LSB	-	3.93 LSB	0.8 LSB
Standard Deviation	6.8 ps	-	-	43 ps	15 ps	21 ps	12.8 ps	-
Frequency of Reference Clock	135.5 MHz	125 MHz	-	100 MHz	120 MHz	400 MHz	600 MHz	710 MHz
